# Meiotic chromosomes in motion: a perspective from *Mus musculus* and *Caenorhabditis elegans*

**DOI:** 10.1007/s00412-019-00698-5

**Published:** 2019-03-15

**Authors:** Jana Link, Verena Jantsch

**Affiliations:** Department of Chromosome Biology, Max F. Perutz Laboratories, Vienna Biocenter, University of Vienna, 1030 Vienna, Austria

**Keywords:** Meiosis, Chromosome movement, LINC complex, Mouse, *C. elegans*

## Abstract

Vigorous chromosome movement during the extended prophase of the first meiotic division is conserved in most eukaryotes. The movement is crucial for the faithful segregation of homologous chromosomes into daughter cells, and thus for fertility. A prerequisite for meiotic chromosome movement is the stable and functional attachment of telomeres or chromosome ends to the nuclear envelope and their cytoplasmic coupling to the cytoskeletal forces responsible for generating movement. Important advances in understanding the components, mechanisms, and regulation of chromosome end attachment and movement have recently been made. This review focuses on insights gained from experiments into two major metazoan model organisms: the mouse, *Mus musculus*, and the nematode, *Caenorhabditis elegans*.

## Introduction

In sexually reproducing organisms, meiotic cell divisions are essential to keep the DNA content constant over generations. During gametogenesis, chromosome replication is followed by two divisions, meiosis I and II, which segregate homologous chromosomes and sister chromatids, respectively. In the first meiotic division, the duplicated parental chromosomes become interconnected through products of homologous recombination, which ensures their faithful segregation and leads to the re-assortment of genetic material. In the extended prophase I of the first meiotic division, the establishment of the tether between the homologous chromosomes (homologs) relies on a series of specific events including chromosome pairing, homologous recombination, and cohesion. The homologs must be sorted, aligned, and paired in a highly organized manner to avoid entanglements or interwoven chromosomes to finally connect them tightly through the synaptonemal complex. At the same time, DNA double-strand breaks (DSBs) are introduced by Spo11, an enzyme with similarities to the A subunit of topoisomerase VI (Keeney et al. 1997). DSB repair via homologous recombination is required to achieve the species-specific number of genetic crossover events, and this process also promotes the formation of the physical connection between parental homologs (for review Zickler and Kleckner 2015). This review will discuss how directed chromosome movements, which are prominent during meiotic prophase I in organism ranging from yeast to mammals (e.g., reviewed in Alleva and Smolikove 2017; Hiraoka and Dernburg 2009; Koszul and Kleckner 2009), contribute to the accurate alignment, pairing, and segregation of homologous chromosomes. We have chosen to focus on two widely used metazoan model organisms: the mouse, *Mus musculus*, and the nematode, *Caenorhabditis elegans*, for several reasons: (1) In both, pronounced chromosome movements occur in meiotic prophase I. This is in contrast to chromosome movement and pairing in Drosophila, which occurs in pre-meiotic cells (reviewed in Cahoon and Hawley 2013; Hughes et al. 2018). (2) Both organisms employ the microtubule network machinery for generating meiotic chromosome movement (Labrador et al. 2013; Lee et al. 2015; Sato et al. 2009; Wynne et al. 2012). (3) *C. elegans* and mouse both have a nuclear lamina underlying the inner nuclear membrane. Yeasts, although widely used meiotic model organisms, lack a nuclear lamina. This last common feature is of particular interest, as components of the nuclear envelope and the lamina are remodeled at meiotic onset, which likely contributes to efficient chromosome movement.

**Fig. 1:**
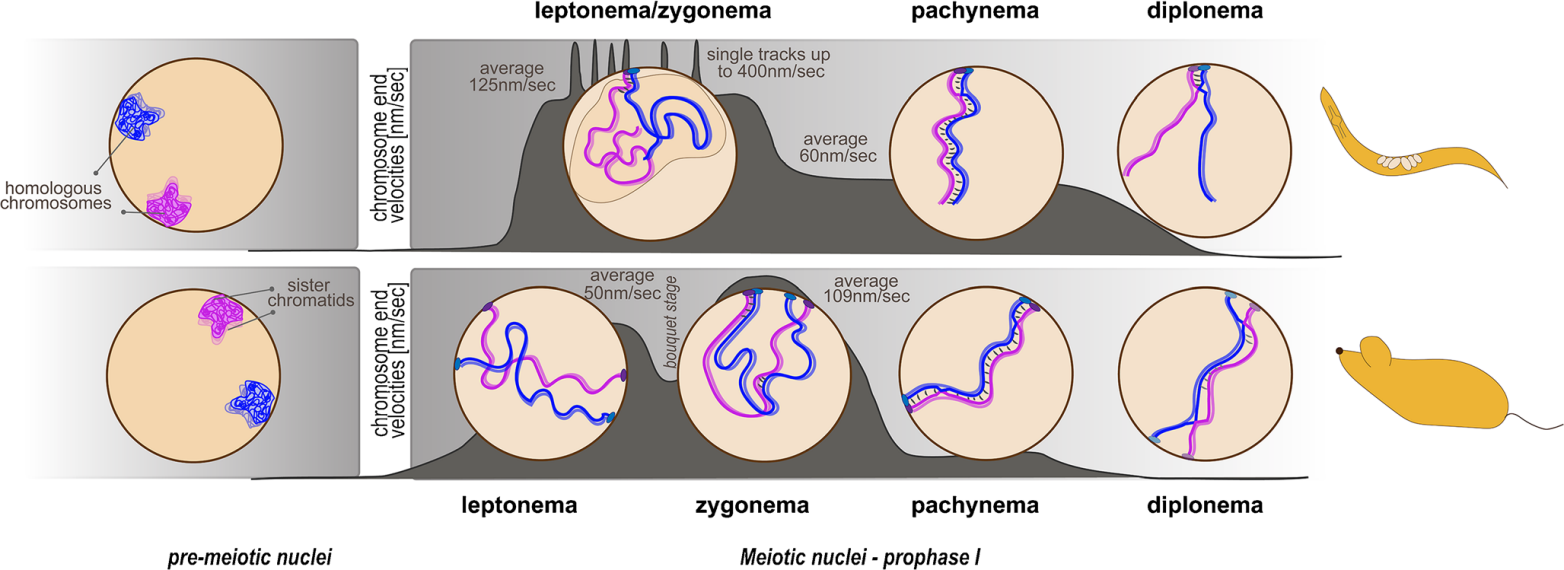
Schematic representation of chromosome dynamics during prophase of the first meiotic division. In *C. elegans* (top panel), one chromosome end is tethered to the nuclear envelope (NE). During leptonema/zygonema, the chromatin clusters at one side of the nucleus. At this stage, rapid prophase movements (RPMs) are most prominent. SUN-1 aggregates move at an average speed of 125 nm/s; however, single highly mobile chromosome ends with a speed of up to 400 nm/s have been measured. Also, synaptonemal complex assembly is initiated at this stage. In pachytene nuclei, synaptonemal complex formation is completed to stably connect homologous chromosomes and allow the single obligate crossover event to occur. At this time, movement of the attached X-chromosome end is still observed, with an average speed of 60 nm/s. During diplotene, the synaptonemal complex is restructured as the central element components retract to the short arm of the bivalent, as defined by the crossover site. In the mouse (bottom panel), both chromosome ends are tethered to the NE in leptonema and active telomere dynamics are observed. Telomere velocities temporarily slow down during the bouquet stage at leptotene/zygotene transition and are maximal during zygonema (average speed 109 nm/s). At this time, the alignment of homologous chromosomes has been established and synaptonemal complex assembly is ongoing. Telomere movement is drastically reduced during pachynema, when the telomeres are again dispersed along the entire NE and homologous chromosomes are fully synapsed. During diplonema, the synaptonemal complex begins to disassemble, with chiasmata indicating crossover sites. Subsequently, telomeres are detached from the NE. Chromosome end velocities for both species are indicated by the dark grey area (*y*-axis: chromosome end velocities in nm/s, drawn not to scale)

Meiotic prophase I comprises the following stages: leptonema, zygonema, pachynema, diplonema, and diakinesis (Fig. 1). During leptonema and zygonema, extensive chromatin reorganization, including widely conserved vigorous chromosome movements (also termed RPM, rapid prophase movements), and nuclear envelope remodeling occur*.* To prepare for the chromosome movements, chromosome ends are tethered and stably attached to the nuclear envelope. In mouse, both telomeres attach to the nuclear envelope at this stage, whereas in *C. elegans*, only one end of each chromosome is tethered to the nuclear envelope by a repetitive sub-telomeric region termed pairing center (Goldstein and Slaton 1982 and reviewed in Woglar and Jantsch 2014). In both organisms attached chromosome ends become connected to the cytoplasmic microtubule cytoskeleton via membrane-spanning protein complexes, thus inducing the vigorous movement (or stirring) of chromosome ends along the nuclear envelope (Lee et al. 2015; Sato et al. 2009). This method of stirring chromosomes within the nucleus using forces generated in the cytoplasm is widely conserved in many organisms ranging from yeasts, plants, and nematodes to mammals (Burke 2018; Zeng et al. 2017). The chromosome movements result in the formation of locally clustered chromosome ends (known as a bouquet), which has been observed cytologically in plants, yeast, and mammals (Chikashige et al. 1994; Dresser and Giroux 1988; Scherthan et al. 1996; Trelles-Sticken et al. 1999). More recent studies have used in vivo imaging to define the bouquet as a transient chromosome configuration occurring at leptonema/zygonema transition (Dresser 2009; Enguita-Marruedo et al. 2018; Lee et al. 2015; Scherthan and Adelfalk 2011; Shibuya et al. 2014g). Although not all functional implications of the meiotic chromosome bouquet have been elucidated so far, the structure seems to contribute to efficient homologous chromosome pairing, in particular by resolving heterologous chromosome interactions (Chacon et al. 2016; Chikashige et al. 2006; Davis and Smith 2006; Tang et al. 2006; and reviewed in Klutstein and Cooper 2014; Zickler and Kleckner 2016). In contrast, *C. elegans* leptotene/zygotene nuclei lack a bouquet; however, in zygonema, the chromatin concentrates at one side of the nucleus and adopts a prominent and characteristic half-moon shape (Dernburg et al. 1998).

Soon after meiotic entry, the chromosome axes connecting the two replicated sister chromatids are assembled onto the chromosome backbones. Thereafter, the first contacts between homologous chromosomes are established and immediately stabilized by the installation of the synaptonemal complex. The exact timing and interdependencies of chromosome pairing, synaptonemal complex assembly, and DSB induction are species specific. In many organisms, including the mouse, induced DSBs by SPO11 are essential for the correct alignment and stable synapsis between homologs (Baudat et al. 2000; Romanienko and Camerini-Otero 2000; for review Zickler and Kleckner 2016). Current models suggest that in organisms in which chromosome pairing and synaptonemal complex formation depend on DSB induction, resected single-stranded DNA fibers generated during homologous recombination act as sensors for detecting homologous sequences (Loidl 1994; Peoples-Holst and Burgess 2005). *C. elegans* differs in that chromosome pairing and synaptonemal complex formation occur in *spo-11* mutants and are thus independent of DSB induction (Dernburg et al. 1998).

By the pachytene stage of mid-prophase, homologous chromosomes are stably paired and connected via the fully assembled synaptonemal complex. During this stage, chromosome ends are redistributed over the entire nuclear envelope, leading to bouquet dissolution (for review Lui and Colaiacovo 2013). Recombination proceeds in parallel with synaptonemal complex installation; indeed, crossover recombination products are often processed in the context of the synaptonemal complex (Jantsch et al. 2004; MacQueen et al. 2002; Woglar and Villeneuve 2018). The processing includes numerous steps such as strand resection to generate 3′ single-stranded overhangs, strand invasion into a sister chromatid of the parental homolog, and second end capture, culminating in the generation of joint DNA molecules, which in many organisms are recognized as double holiday junctions. The joint DNA structures are later dismantled and some recombination products are resolved as genetic crossover events. The crossovers can be seen cytologically as chiasmata at diplonema, once the synaptonemal complex begins to disassemble (for review Hunter 2015). Chromosome restructuring and condensation continue throughout diplonema and diakinesis, when condensed separated chromatin bodies can be observed. These structures represent the paired bivalents of each chromosome pair connected by crossover(s). The establishment of at least one crossover per homologous chromosome pair is essential for their correct alignment on the metaphase plate and subsequent faithful partitioning into daughter cells by the spindle apparatus.

In the past years, numerous studies have provided detailed insight into various aspects of chromosome movement in meiotic prophase in different model organisms, from describing the phenotypes that result from abrogation of chromosome movement to the regulation of chromosome end attachment to the nuclear envelope. Here, we summarize the insights on two metazoan model systems, which both take advantage of the microtubule cytoskeleton to drive chromosome movements in early meiosis (Fig. 1).

## The machinery involved in chromosome end attachment and movement

In both the mouse and *C. elegans*, chromosome mobility relies on the microtubule cytoskeleton and microtubule-associated dynein is necessary for chromosome movements (Labrador et al. 2013; Lee et al. 2015; Sato et al. 2009; Wynne et al. 2012). Chromosome movement during prophase I is abolished in both organisms in the presence of microtubule inhibiting or depolymerizing drugs (Sato et al. 2009; Shibuya et al. 2014g; Wynne et al. 2012). In mouse spermatocytes, microtubule tracks/cables running along the cytoplasmic surface of the nuclear envelope have additionally been observed by cytology (Lee et al. 2015; Shibuya et al. 2014g). These microtubule filaments extend outward from the nucleus into the microtubule network in the cytoplasm. Whilst the microtubule cables surrounding the nuclear envelope are not in any particular relationship with the microtubule-organizing center, they differ in bundle thickness and arrangement depending on the stage of prophase I (Lee et al. 2015). Furthermore, components of the force-transducing LINC complex residing in the nuclear envelope (see below) partially co-localize with the observed microtubule cables. This substantiates the hypothesis that this sub-population of microtubule filaments is involved in the RPMs. The actin cytoskeleton is dispensable for chromatin reorganization in early *C. elegans* meiosis (Sato et al. 2009) but seems to contribute to nuclear shape in mouse meiotic nuclei (Shibuya et al. 2014g) and to gonad architecture in *C. elegans* (Zhou et al. 2009).

**Fig. 2:**
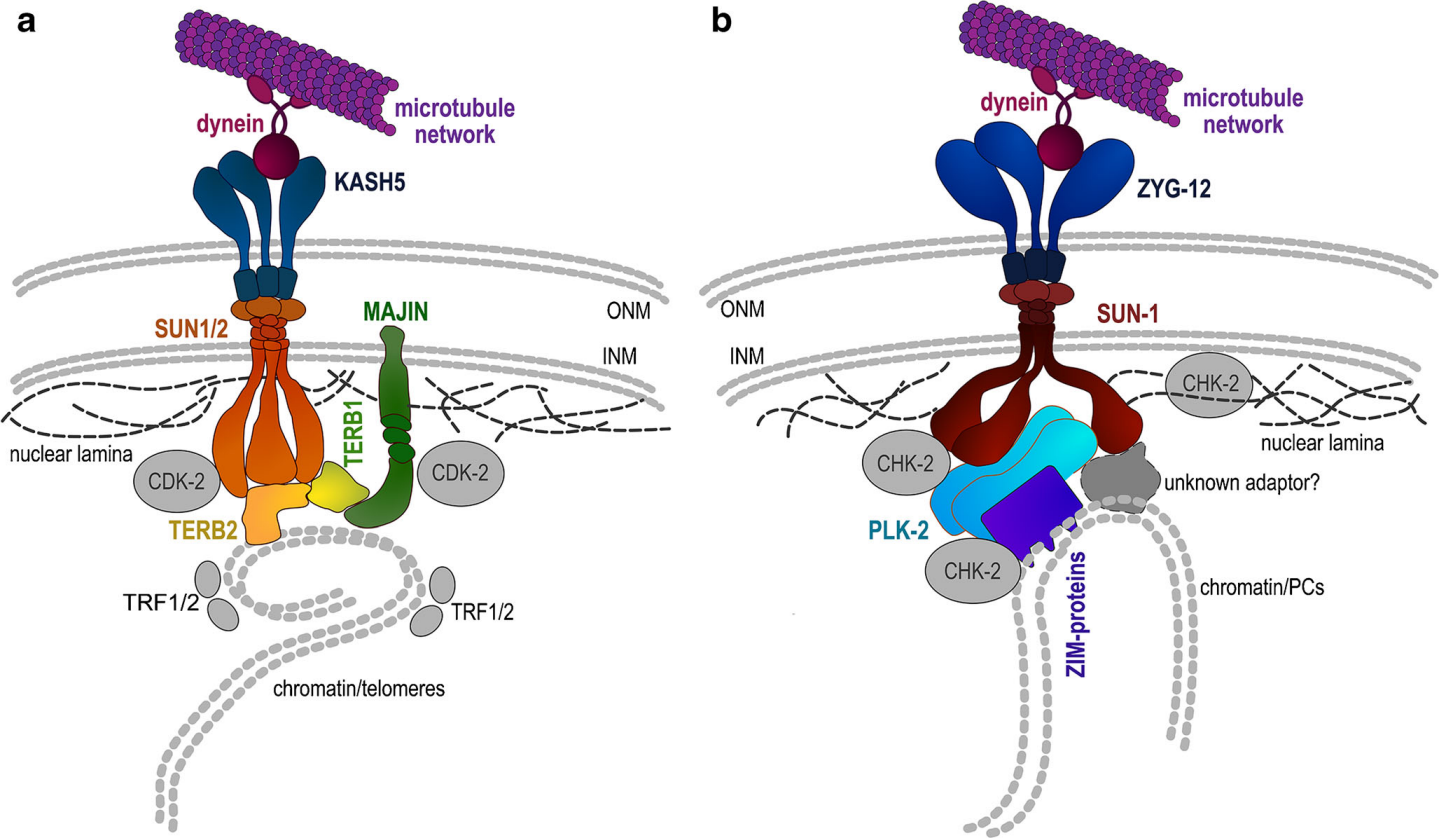
Schematic representation of the machinery involved in meiotic chromosome-end attachment and movement in the mouse (**a**) and in *C. elegans* (**b**). **a**) After cap exchange, the mature telomere-attachment complex in mouse comprises TERB1, TERB2, and MAJIN. TERB2 and MAJIN can both interact directly with telomeric DNA, thus mediating the interaction between the telomere and SUN1. The LINC complex itself, connecting to dynein and the microtubule network, consists of SUN1/2 in the inner nuclear membrane (INM) and KASH5 in the outer nuclear membrane (ONM). TRF1 is displaced from the actual site of telomere attachment during cap exchange and is replaced by MAJIN in interacting with telomeric DNA. CDK activity is implicated in regulating cap exchange and perhaps also stability of the SUN1–KASH5 interaction or meiotic membrane integrity. **b**) In *C. elegans*, the pairing centers (PCs), located in the subtelomeric region of one chromosome end, interact with the PC zinc-finger proteins. The PCs serve as recruitment sites for PLK-2, which is primed by PC protein phospho-modifications by the CHK-2 kinase. PLK-2 recruitment correlates with SUN-1 aggregation, and known substrates are SUN-1 and LMN-1. Additional, yet unidentified, adaptor proteins involved in coupling SUN-1 to chromatin or to ZIMs may exist. SUN-1 interacts with ZYG-12 in the ONM, which in turn connects to dynein, which mediates the interaction with the microtubule network

Forces generated in the cytoplasm are transduced by the LINC (linker of nucleoskeleton and cytoskeleton) complex through the nuclear membranes to chromosome ends attached at the nuclear periphery (Crisp et al. 2006). The LINC complex spans the nuclear membranes, consisting of KASH-domain proteins (Klarsicht, Syne homology) in the outer nuclear membrane and SUN-domain proteins (Sad1 UNC domain) in the inner nuclear membrane (Fig. 2). In mouse, ubiquitously expressed SUN1 and SUN2 proteins are evenly distributed on the nuclear rim in pre-meiotic cells. Upon meiotic onset, they relocate within the nuclear envelope into aggregates that colocalize with attached telomeres to form the meiotic LINC complex together with KASH5 (Ding et al. 2007; Horn et al. 2013; Link et al. 2014; Morimoto et al. 2012; Schmitt et al. 2007) (Fig. 2a). *C. elegans* SUN-1 is exclusively expressed in the germline and embryos, where it forms a meiosis-specific LINC complex with its KASH partner ZYG-12 (Fig. 2b). ZYG-12 is anchored to the outer nuclear membrane by SUN-1, whereas SUN-1 nuclear rim localization occurs also in the absence of ZYG-12 (Penkner et al. 2007). Shortly after meiotic entry, SUN-1 redistributes to become concentrated at chromosome end attachment sites (Penkner et al. 2009; Sato et al. 2009). Whereas SUN-domain proteins involved in meiotic LINC-complex formation in mouse localize exclusively to sites of telomere attachment (Ding et al. 2007; Schmitt et al. 2007), in *C. elegans*, SUN-1 localizes within the entire nuclear rim and additionally enriches within mobile aggregates at sites of chromosome end attachment (Penkner et al. 2009). Additionally, the populations of SUN-1 within the rim and within the mobile aggregates are phospho-modified (Baudrimont et al. 2010; Penkner et al. 2009; Woglar et al. 2013). Some modifications are found on the entire population of SUN-1, and others are exclusively found at the SUN-1 molecules forming the aggregates at the chromosome end attachments. How these, potentially separate, populations of SUN-1 are involved in chromosome end attachment in the worm still remains to be elucidated.

The mechanism through which telomeres are connected to the meiotic LINC complex is particularly well studied in the mouse (reviewed in Shibuya and Watanabe 2014). A combination of cytological, genetic, and biochemical studies has shown that a meiosis-specific telomere complex remodels the somatic shelterin complex. The shelterin complex is a multiprotein assembly required for telomere end protection, consisting of TRF1, RAP1, TIN2, TPP1, and POT1 (de Lange 2005). The central components responsible for meiotic telomere restructuring are TERB1/TERB2 and MAJIN (membrane-anchored junction protein) (Daniel et al. 2014; Shibuya et al. 2015, 2014a). TERB1/2 (telomere repeat-binding bouquet formation proteins 1 and 2) are meiosis-specific telomere-binding proteins that associate with telomeres upon meiotic onset. TERB1 consists of a coiled-coil domain with N-terminal armadillo repeats and a C-terminal DNA-interaction motif. The armadillo repeats probably mediate protein–protein interactions, while the conserved C-terminal telobox consensus sequence interacts with telomeric DNA sequences (Daniel et al. 2014). Within the TERB1/2–MAJIN complex, TERB1 acts as the central hub of the meiotic telomere cap through its associations with the telomere-binding protein TRF1 (telomeric repeat binding factor 1), TERB2, and cohesin via distinct interaction domains (Long et al. 2017; Zhang et al. 2017). MAJIN is a meiotic transmembrane protein with a single C-terminal transmembrane domain and a DNA-binding domain, with the latter allowing direct interaction with telomeric DNA (Shibuya et al. 2015).

TERB1 is involved in the assembly of two functionally distinct complexes. The first, the TRF1–TERB1–TERB2–MAJIN–SUN1–KASH5 complex, is essential for telomere-membrane tethering and chromosome end attachment via the telomere. Its interaction with TRF1 is furthermore critical for SUN1 recruitment to the tethered telomeres (Long et al. 2017). As demonstrated in studies using knockout mouse strains, the absence of any one of the complex components severely impairs or prevents telomere attachment (Ding et al. 2007; Horn et al. 2013; Long et al. 2017; Shibuya et al. 2014a; Wang et al. 2018; Zhang et al. 2017). The second, the TRF1–TERB1–cohesin complex, appears necessary to maintain telomere rigidity and integrity during chromosome movement. Within this subcomplex, TERB1 interacts directly with the meiosis-specific cohesin subunit SA3 (Shibuya et al. 2014a). Both nuclear envelope attachment and structural integrity of telomeres are essential for RPMs.

Structural, stoichiometric, and mechanistic insights into meiotic telomere attachment to the nuclear periphery in mouse were derived from detailed studies of the MAJIN-TERB2-TERB1-TRF complex (Dunce et al. 2018; Long et al. 2017; Wang et al. 2019). The crystal structures of MAJIN-TERB2 and TERB2-TERB1 subcomplexes have led to the identification of essential amino acid residues, which mediate the interactions within the telomere complex. Furthermore, mutating these residues disrupt the interactions in vivo, leading to defects in telomere attachment and thus infertility (Wang et al. 2019). Separate analysis of the TRF1 interaction surface of TERB1 at the single amino acid level has shown that TERB1 adopts a similar strategy to bind TRF1 as to bind the TIN2 (TRF1-interacting nuclear protein 2) shelterin component, its usual interaction partner. This binding region contains a predicted CDK phosphorylation site, and a phospho-mimetic mutation interferes with the interaction to TRF1. This mechanism confers reversibility to TERB1 binding by TRF1, enabling TRF1 to disassociate from the TERB1/2–MAJIN complex later in prophase I. The maturation process of the meiotic telomere complex is known as cap exchange (Long et al. 2017; Pendlebury et al. 2017; Shibuya et al. 2015). During cap exchange, bound telomeric DNA is transferred from the displaced shelterin component TRF1 to MAJIN. The MAJIN-TERB2-TERB1 complex recruits telomere-bound TRF1 dimers, thereby tethering telomeres to the nuclear envelope (Dunce et al. 2018). The binding of TRF1 to the meiotic telomere complex inhibits the direct DNA interaction of MAJIN and TERB2 within the complex. Once TRF1 is displaced from the complex, the DNA-binding capacity of MAJIN and TERB2 is restored, facilitating the direct interaction of telomeric DNA with the mature meiotic telomere cap complex (Dunce et al. 2018). The displacement of multiple shelterin components from the actual site of telomere attachment can also be observed cytologically, as in pachytene, the shelterin components form a dissociated ring-like structure in the area surrounding the TERB1/2–MAJIN complex (Dunce et al. 2018; Shibuya et al. 2015). Further investigation of the TERB1–TRF1 interaction surface demonstrated that while disrupting the interaction impairs telomere attachment, also artificially reinforcing the interaction interferes with the cap exchange (Pendlebury et al. 2017). This finding suggests that changes in telomeric protein–protein interaction affinities must be tightly regulated during prophase I, potentially by CDK-dependent phosphorylation. Furthermore, meiosis-specific knockout experiments revealed that TRF1 also plays a role in telomere end protection during early prophase, when chromosome movements take place. One model proposes that TRF1-dependent telomere end protection is conferred through CDK2 recruitment via Speedy A and the conventional shelterin complex (Wang et al. 2018).

Deletion of the meiosis-specific cohesin SMC1beta revealed a distinct role of cohesins in telomere attachment and integrity in the mouse. In the absence of SMC1beta, telomere tethering and the stability of telomere attachment are impaired (Adelfalk et al. 2009; Biswas et al. 2018; Herran et al. 2011). The function of SMC1beta in promoting the complete and stable attachment of telomeres to the nuclear envelope is separable from its function in connecting sister chromatids. SMC1beta is also critical for SMC3 recruitment to the attached telomeres and for protecting the telomere structure (Adelfalk et al. 2009). Maintaining telomere structure is critical during meiosis, as it has been shown that reduced telomere length leads to a defect in meiotic telomere attachment (Liu et al. 2004). The localization of additional meiosis-specific cohesin complex components such as REC8 and RAD21L also extends from the axes to the sites where chromosome ends are attached to the nuclear rim. In the absence of TERB1, deposition of these cohesin components onto chromosome ends is strikingly reduced (Shibuya et al. 2014a). Cohesin recruitment to the TRF/TERB complex is also required for efficient telomere movement, either for mechanical stability or for transducing the force to the attached telomeres (Zhang et al. 2017).

Telomere attachment sites in the mouse have additionally been delineated using high-resolution imaging (Adelfalk et al. 2009; Liebe et al. 2004; Schmitt et al. 2007; Shibuya et al. 2015; Viera et al. 2015). Tethered telomeres form part of an attachment plate structure, where conical thickening of chromosome ends toward the nuclear envelope is observed. It is not yet fully elucidated whether the deposited cohesins are a structural component of the telomere attachment plate.

In *C. elegans*, chromosome coupling to the nuclear periphery is mediated by subtelomeric chromosome regions called homology recognition regions or pairing centers (PCs) (Fig. 2). PCs are composed of short repetitive sequences and have been mapped to one end of each chromosome, thus coupling only one chromosome end to the nuclear envelope (Herman and Kari 1989; McKim et al. 1988, 1993; Phillips et al. 2009; Rose et al. 1984; Rosenbluth and Baillie 1981; Sanford and Perry 2001; Villeneuve 1994). PCs are necessary for the stable pairing, alignment, and synapsis of homologous chromosomes, probably through their central role in chromosome end attachment (MacQueen et al. 2005; Woglar and Jantsch 2014). Four members of a family of zinc-finger proteins localize to PCs at the nuclear envelope and are essential for coupling chromosome ends to the LINC complex (Phillips and Dernburg 2006; Phillips et al. 2005). Each member of this zinc-finger protein family is responsible for binding to the PCs of specific chromosomes: ZIM-1 to chromosomes II and III, ZIM-2 to chromosome V, ZIM-3 to chromosomes I and IV and HIM-8 to the X chromosome (Phillips and Dernburg 2006). Simultaneous immunofluorescence labeling of all PC proteins shows that they colocalize strongly with SUN-1 aggregates, which also supports the notion that PCs are essential to transfer cytoplasmic forces onto chromosomes via the LINC complex (Sato et al. 2009). Unlike in the mouse, nothing is known about the structural components or ultrastructural features of attachment plates in *C. elegans*. Most importantly, the factor(s) directly connecting chromosome ends to the LINC complex partner SUN-1 remain unidentified. In mutants deficient for components of the meiotic LINC complex (SUN-1/ZYG-12), PC proteins still seem to associate with the nuclear rim. Direct interaction between SUN-1 and PC proteins has not been demonstrated (Sato et al. 2009). These observations suggest that unknown components or distinct protein modifications of SUN-1 and/or PC proteins may be involved in chromosome end tethering in *C. elegans*. Whether telomeres themselves also play a role in chromosome attachment to the nuclear periphery remains to be clarified.

## Regulation of chromosome end attachment and coupling to the movement apparatus

Chromosome end attachment to the movement apparatus exclusively occurs at meiotic onset and must therefore be tightly regulated. The regulation of tethering, attachment, and movement of chromosome ends is thus a matter of intense investigation. Work in *C. elegans* established the regulatory roles of kinases in chromosome movement and their linkage to meiotic progression. CHK-2, the homolog of mammalian Chk2, was identified as a meiotic master regulator, as *chk-2* mutants lack meiotic nuclear reorganization and pairing, both features of chromosome end-led mobilization (MacQueen and Villeneuve 2001). Furthermore, CHK-2 kinase activity is also essential for the phosphorylation and aggregate formation of SUN-1 (Penkner et al. 2009). Major meiotic regulatory functions have also been demonstrated for Polo kinases, which are conserved members of the polo-like family of Ser/Thr kinases. *C. elegans plk-2* mutants lack chromosome mobilization, SUN-1 aggregation at the chromosome end attachments and homologous pairing (Harper et al. 2011; Labella et al. 2011). PLK-2 accumulates at PCs associated with the nuclear envelope at meiotic onset, colocalizing with SUN-1/ZYG-12 aggregates (Labella et al. 2011). PCs and their associated proteins have a pivotal role in regulating chromosome end-led motions. In the absence of PC proteins, achieved through deletion of the encoding operon, chromosome clustering and movement, as well as SUN-1/ZYG-12 aggregate formation, are absent (Harper et al. 2011). The initial recruitment of most PC proteins and PLK-2 is dependent on CHK-2 kinase activity; thereby, CHK-2 fulfills the function of a priming kinase acting on the polo box domain within the PC proteins (Harper et al. 2011; Kim et al. 2015; Phillips and Dernburg 2006). Once PC proteins relocate to the subtelomeric chromosomal regions, they colocalize with the nuclear periphery and recruit PLK-2 (Labella et al. 2011). If functional PLK-2 is lacking at the PC, SUN-1 in turn is not phosphorylated on specific residues and does not form aggregates (Harper et al. 2011; Labella et al. 2011). PC proteins are essential for the local enrichment of kinases that mediate chromosome end mobility (Harper et al. 2011). In addition, the PCs also regulate or deliver kinase activity toward other nuclear envelope components, such as the nuclear lamina (Link et al. 2018). The duration of chromosome movement in *C. elegans* is regulated by a feedback loop involving the same kinase cascade and the phosphorylation of SUN-1, which is required for persistent chromosome movement (Woglar et al. 2013). Incomplete synapsis formation or a failure to produce crossover intermediates extends SUN-1 phosphorylation in a PLK-2/CHK-2-dependent manner, prolonging active chromosome movement and the zygotene stage. The SUN-1 phosphorylation itself is in turn required to prolong PLK-2 localization at chromosome ends to extend the period of movement.

Regulation of chromosome end attachment and movement through phospho-modifications seems to also occur in mouse, since evidence for regulatory functions of CDK2 in meiotic telomere attachment and movement is accumulating. A meiosis-specific non-canonical CDK activator, Speedy A, is essential for the complete and functional tethering of telomeres to the nuclear periphery. The N-terminal domain of Speedy A binds to CDK2 and is essential for telomere attachment and CDK2 recruitment to the attachment sites (Mikolcevic et al. 2016; Tu et al. 2017). TRF1 interacts with Speedy A and is thus likely to directly recruit CDK2 to telomeres, a process needed for telomere end protection (Wang et al. 2018). In Speedy A knockout mice, the TRF1 signal is lost from the nuclear rim, suggesting that CDK2 has an active role in the nuclear envelope tethering of TRF1 (Tu et al. 2017). The observation of CDK2 localization to telomeres associated with the nuclear envelope at the attachment plates led to studies into the consequences of CDK2 deficiency in meiosis (Viera et al. 2015; Viera et al. 2009). Multiple roles for CDK2 in early prophase I were identified: Mutants had defective telomere tethering, along with telomere fusions and overall disorganization of the nuclear envelope and its associated proteins. In the absence of CDK2, essential players such as SUN1, KASH5, and lamin C2 form a cap-like structure adjacent to the centrosome instead of redistributing at meiotic onset as they do in the wild type. Interestingly, the dislodged telomeres are coupled to membrane-associated vesicles, suggesting that they have been ripped from the membrane. Based on these results, the authors hypothesize that the aberrantly assembled synaptonemal complex in the mutant exerts tension on the chromosome end attachments and that a lack of CDK2 would cause telomeres to be released from the nuclear envelope. Although CDK2 can phosphorylate SUN1 in vitro, it remains to be shown whether SUN1 is the physiological target of CDK2 during prophase I (Viera et al. 2015).

Kinase activities are not the only regulators of chromosome attachment and movement. Altered duration or features of RPMs also correlate with changes in meiotic progression in both mouse and worm, suggesting a cross talk between meiotic recombination intermediates, chromosome structure, and meiotic chromosome movements. In particular, the availability of various genetic backgrounds and the convenient accessibility to in vivo imaging techniques has allowed the identification of several factors that influence chromosome movement characteristics in *C. elegans*. Besides the essential components, such as CHK-2 and polo kinase activity and an intact SUN–KASH bridge, chromosome lateral element formation is also needed for wild-type-like SUN-1 aggregate movement (Baudrimont et al. 2010; Wynne et al. 2012). Mutant backgrounds in which lateral element formation is abrogated show reduced SUN-1 aggregate velocities and distances traveled as well as a reduced dynamic in splitting and fusion events or overall reduced SUN-1 aggregate formation (Penkner et al. 2009; Baudrimont et al. 2010). Also in mice, synapsis and recombination intermediates have been reported to influence chromosome movement characteristics (Lee et al. 2015). Mouse mutants defective in synaptonemal complex assembly or early/mid recombination intermediates show significantly reduced chromosome end velocities (Lee et al. 2015). Furthermore, *C. elegans spo-11* mutants, defective in DSB formation, show altered SUN-1 aggregate movement indicating that the induction of DSBs is also critical for wild-type chromosome mobility (Baudrimont et al. 2010; Machovina et al. 2016). Similarly, in mouse, DSB induction and ATM-dependent DSB signaling correlate with increased bouquet frequencies in the mutants indicating reduced telomere dynamics (Liebe et al. 2006). However, the mechanism linking DSB induction, signaling, and repair to active chromosome movement remains to be determined in either organism. These data from both organisms indicate that meiotic events occurring along the chromosomes are likely able to signal to and influence attached chromosome end movement.

It is becoming increasingly clear that also components of the nuclear envelope, which are not directly involved in telomere tethering or attachment, influence and regulate meiotic chromosome dynamics. In both mice and worms, the nuclear lamina is modulated in a meiosis-specific manner. In mouse, this is achieved by expressing the meiosis-specific lamin C2 as the only A-type lamin isoform, in addition to lamins B1 and B2 (Alsheimer and Benavente 1996; Link et al. 2013). In *C. elegans*, the single lamin protein is specifically phosphorylated upon meiotic entry, which contributes to opening the rigid lamina network (Link et al. 2018). Although the meiotic consequences in male mice are more drastic, modulation of the nuclear envelope is necessary for efficient chromosome movement and meiotic progression in both model organisms.

## Characteristics of meiotic chromosome movements

Observations of chromatin oscillations and nuclear rotations during prophase have already been made decades ago in rodents. Using phase contrast time-lapse microscopy, spermatocytes in cultured seminiferous tubules of Chinese hamsters and rats were investigated (Ellingson and Yao 1970; Parvinen and Soderstrom 1976; Yao and Ellingson 1969). It was already observed then that nuclear rotations and chromatin oscillations were most active during early zygonema, slowed down toward the end of zygonema and ended during pachynema (Parvinen and Soderstrom 1976). Before it was known that the LINC complex mediates force transmission, cytoskeletal forces were suggested to drive the movements. In accordance with this, nuclear movements were completely abrogated in cultured spermatocytes treated with colcemide to inhibit microtubule formation (Salonen et al. 1982). The coincidental timing of nuclear rotations and synapsis formation was indicative of a correlation between homolog pairing and directed nuclear movements (Parvinen and Soderstrom 1976). Most analyses to understand the pairing behavior of chromosomes were, however, limited to fixed samples, especially when combined with fluorescent in situ hybridization (Cobb and Handel 1998; Scherthan et al. 1996). Thus, chromosome dynamics, in particular the clustering of telomeres within the nuclear envelope, could only be deduced from observations of sequential images at different stages of prophase I. Advances in fluorescence in vivo imaging have improved the direct observation of meiotic chromosome movements in both the mouse and *C. elegans*, leading to a more detailed characterization of these RPMs.

In male mice, meiosis occurs in the seminiferous tubules in the testes, where they are hard to access for live imaging. To circumvent this, explanted seminiferous tubules can be cultured and spermatocytes can be imaged within the tubules or in cellular suspension. Due to the presence of all meiotic stages within the tubules, both approaches require accurate cell staging to distinguish different sub-stages of prophase I. Changes in pericentric heterochromatin and major satellite DNA patterns were used for both staging mouse spermatocytes and deducing chromatin mobility within the nuclei (Lee et al. 2015; Scherthan et al. 1996). More recent studies used fluorescently tagged components of the synaptonemal complex to stage spermatocytes more accurately (Enguita-Marruedo et al. 2018; Morelli et al. 2008; Shibuya et al. 2014g). A detailed quantification of mouse RPMs was first performed by Watanabe and colleagues (Morimoto et al. 2012; Shibuya et al. 2014g), and was quickly followed by more mechanistic investigations (Lee et al. 2015). Lee et al. performed imaging on explanted cultured seminiferous tubules, which might reflect the more physiological situation in the intact tissue compared to the spermatocyte cell suspension used by Watanabe and colleagues. They, however, transiently transfected fluorescently tagged TRF by electroporation (Shibuya et al. 2014g), which allowed the direct visualization and quantification of telomere movement, in contrast to following heterochromatin spots over time. The direct visualization of the labeled moving telomeres revealed finer details in motion. Nonetheless, both studies concur that active chromosome movements occur throughout prophase I but are regulated in a substage-specific manner. Additionally, individual chromosome movements can be distinguished from concerted chromosome movements and nuclear rotations. Lee et al. computationally separated nuclear rotations or concerted movements from single telomere movements, thus calculating the “residual” velocity of single spots (Lee et al. 2015). Whereas pre-meiotic S-phase nuclei have very limited chromosome mobility, leptotene/zygotene nuclei have average chromosome end velocities of 109 nm/s (Lee et al. 2015) or 120 nm/s (Shibuya et al. 2014g). RPMs are slower during the bouquet stage at the leptotene/zygotene transition and peak during zygonema. Lee et al. specifically measured the average speed of movement before, during, and after the bouquet formation and found that it was only 28.5 nm/s during the bouquet stage (Lee et al. 2015). Furthermore, only telomeres engaged in a bouquet configuration show a reduced mobility, whilst non-clustered telomeres were still highly mobile within the same cell (Shibuya et al. 2014g). Thus, the reduced mobility observed during the bouquet seems to be regulated on a single-telomere level. Chromosome movements are reduced in pachytene or diplotene nuclei.

**Fig. 3:**
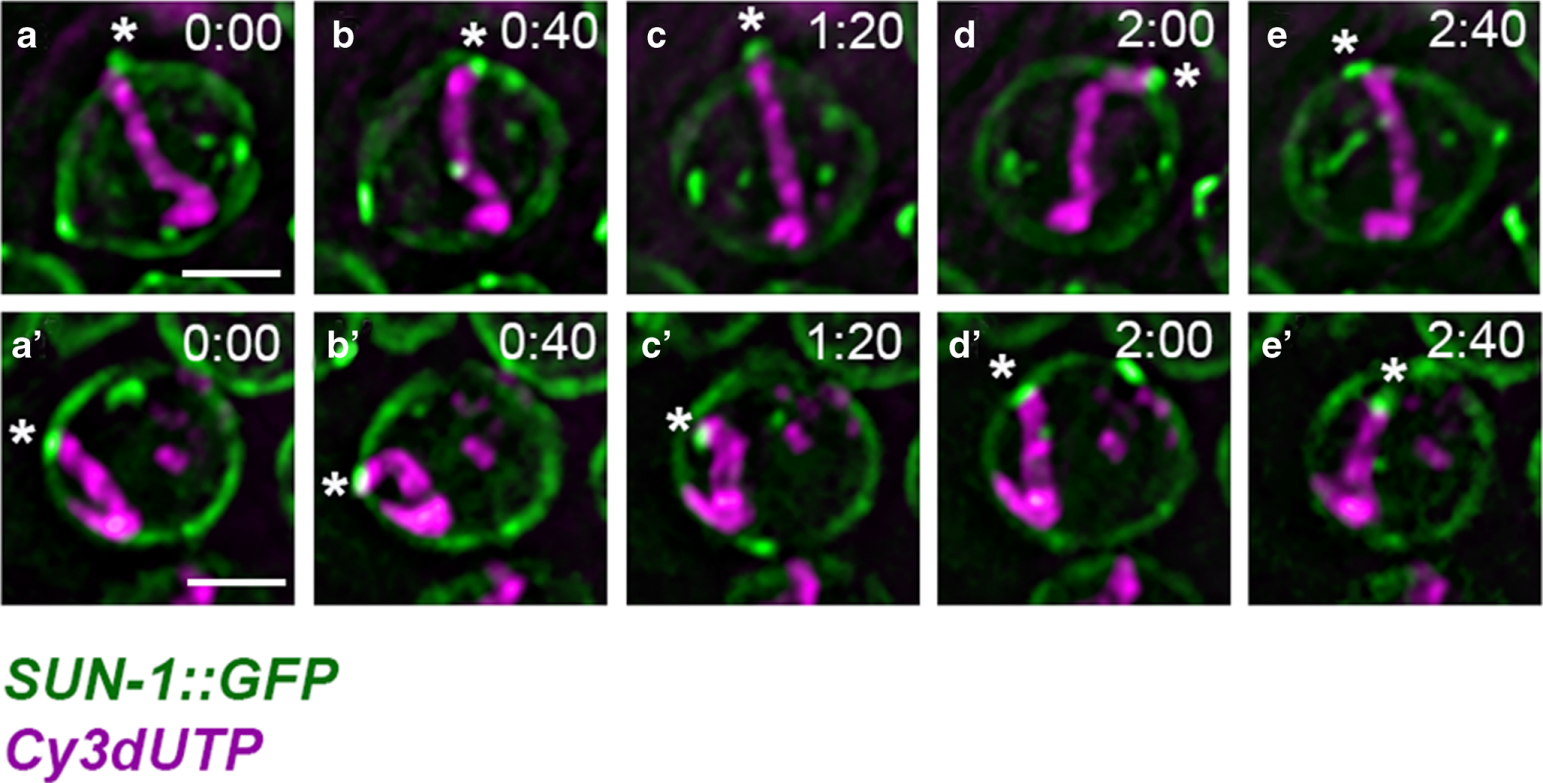
Time-lapse images of Cy3-dUTP-labeled X-chromosomes in two leptotene/zygotene nuclei (**a**–**e**; **a′**–**e′**). Live imaging of Cy3-dUTP-labeled X-chromosomes (magenta) in *sun-1::gfp* (green) worms reveals that the attached chromosome end, which colocalizes with SUN-1 aggregates, moves vigorously within the nuclear envelope, while the remainder of the labeled chromosome remains relatively static. In some nuclei, a large proportion of non-paring center (PC) chromosome ends seems to be in contact with the nuclear periphery (**a′**–**e′**). The asterisks indicate the mobile chromosome end that is attached to the nuclear envelope by the PC. Time stamps are shown in min/s. Scale bar 2 μm

*C. elegans* is transparent throughout the life cycle and easily anesthetized, with a range of fluorescently tagged transgenes available to monitor chromosome structures over time. These properties have made *C. elegans* a suitable animal model to elucidate meiotic chromosome movements in detail in the live animal under physiological conditions. In addition, the well-defined spatiotemporal progression of meiosis within the gonad enables fast, accurate staging of prophase I nuclei. In vivo imaging of fluorescently tagged proteins involved in chromosome end attachment has provided detailed insight to stage-specific chromosome dynamics (Baudrimont et al. 2010; Wynne et al. 2012). As in the mouse, *C. elegans* pre-meiotic nuclei have limited chromosome mobility (Wynne et al. 2012). Soon after meiotic entry, the chromosome ends attached to the nuclear envelope become highly mobile, whereas other chromosome regions remain more static (Fig. 3) (Wynne et al. 2012). The apparatus responsible to move chromosome ends, SUN-1 and ZYG-12 together with PC proteins, forms highly mobile aggregates at the attachment sites. Those aggregates, which can be imaged, move separately but have a high tendency to come together into groups or clusters. Although the clustered chromosome ends show concerted movements, individual chromosome ends can join or leave these cluster. The chromosome ends generally remain associated for around 1–3 min, although some clusters have been observed to persist for over 3 min (Baudrimont et al. 2010; Penkner et al. 2009; Sato et al. 2009; Wynne et al. 2012). The FKB-6 chaperone, which is localized in the cytoplasm, increases the resting time of individual aggregates within a group/cluster, thus increasing the dynamics of fusion/splitting events in aggregates in the mutant. This observation suggests that the movements may be actively, temporally downregulated when chromosome ends come together in clusters. Because the formation of the synaptonemal complex is delayed in the *fkb-6* mutant (Alleva et al. 2017), the temporally reduced movement could enable the synaptonemal complex to be established between homologs. Although Lee et al. also observed merging and separating of heterochromatin spots in the mouse, this is not comparable with the dynamics of aggregate fusions and splitting observed in *C. elegans*, which occur at high frequency. Nuclear rotations, as in the mouse, were not observed in worms. However, high-resolution three-dimensional analysis of chromosome ends showed that in *C. elegans*, single processive movements are superimposed by undirected movements in small steps, appearing almost as oscillations on the spot. Wynne et al. was able to separate these two modes of movement using high-speed imaging at 400-ms intervals. Chromosome ends have been reported to move at 40–160 nm/s (Baudrimont et al. 2010) or at an average speed of 125 nm/s (Wynne et al. 2012), depending on the imaging conditions. Exceptionally fast saltatory tracks have also been observed, with a maximum velocity of 400 nm/s. Such chromosome end trajectories can be as long as 2 μm; however, they are 0.5 μm on average (Wynne et al. 2012). Despite their rarity, these fast trajectories are responsible for most of the exploration of the nuclear surface area by chromosome ends. Interestingly, the characteristics of chromosome end movement (i.e., speed, fusion and splitting events) remain constant throughout zygonema despite the continuous pairing and synapsis progress (Baudrimont et al. 2010). In *C. elegans*, RPMs are not restricted to leptotene and zygotene nuclei: X chromosome movement has also been observed in pachynema.

Overall, the two model organisms share some similar features of movement. Both worm and mouse show solitary chromosome end movement, and there is evidence that movement may also be regulated on a single-chromosome basis (Machovina et al. 2016; Shibuya et al. 2014g). In both organisms, groups of chromosome ends move together and the constellation of these groups seems to be rather dynamic, particularly in *C. elegans*. Furthermore, movement characteristics (directionality, speed, and movement duration) are consistent with chromosome ends being dragged along microtubule cables (Lee et al. 2015; Shibuya et al. 2014g; Wynne et al. 2012). Thus, the microtubule network plays an essential role in both organisms, and nuclear envelope associated microtubule cables are prominently observed in the mouse. Speed ranges of chromosome end movement are comparable in the two organisms with an average velocity of 109–120 nm/s in mouse zygotene spermatocytes versus an average velocity of 125 nm/s in *C. elegans* zygotene nuclei (Alleva and Smolikove 2017). Exclusive features of mouse prophase nuclei are the dominant nuclear rotations and strong concerted chromosome movements of heterochromatin clusters. In *C. elegans*, on the other hand, SUN-1 aggregates (delineating the chromosome end attachments) are more variable in size, which reflects their dynamic associations into groups. *C. elegans* SUN-1 aggregates, therefore, seem to build up “variable chromosome end clusters” with frequent splitting and fusion events.

## Functions of directed chromosome movement

Originally, it was assumed that the sole purpose of meiotic RPMs and the concomitant meiotic bouquet was to facilitate meiotic pairing by bringing homologous chromosomes into proximity. However, work in the mouse has shown that this might be an oversimplification because partial homolog recognition and association can occur upon meiotic entry, before DSB induction, axis assembly, or telomere clustering (Boateng et al. 2013; Ishiguro et al. 2014). Nonetheless, a mutant mouse expressing TERB1 with a perturbed TRF1 interaction surface clearly showed that chromosome movement assists pairing, at least for sex chromosomes (Long et al. 2017). This mutant has a striking sexually dimorphic phenotype leading to male infertility caused by an inability to pair the short pseudoautosomal region of X and Y chromosomes.

Genetic evidence indicates that chromosome movement may also facilitate efficient homologous pairing as well as faithful synapsis formation in *C. elegans*. The expression of the *sun-1(jf18)* allele, which abrogates SUN-1 binding to its KASH partner ZYG-12, leads to extensive heterologous synapsis and fold-back synapsis, where chromosomes aberrantly synapse with themselves (Penkner et al. 2007; Rog and Dernburg 2015). In this mutant, chromosome ends are efficiently tethered to the nuclear envelope and yet are not moved by cytoskeletal forces (Baudrimont et al. 2010), suggesting that movement is necessary for efficient homologous pairing. A similar phenotype was observed in *spd-3* mutants, which also show reduced chromosome end movement and extensive heterologous synapsis (Labrador et al. 2013). SPD-3, a mitochondrial protein, might be involved in generating the energy needed for movement. In worms, the prevention of heterologous synapsis (often fold-back self-synapsis) by chromosome movement thus clearly promotes pairing between homologous chromosomes (Rog and Dernburg 2015). It has been suggested that homologs would encounter each other by diffusion alone if non-homologous contacts were not fixed by non-homologous synapsis (Wynne et al. 2012). However, specific chromosomal loci do not pair when synapsis is blocked in movement-deficient genetic backgrounds, suggesting that besides enforcing proper synapsis, movement also helps to ensure efficient pairing (Penkner et al. 2007).

Depletion of either ZYG-12 or dynein in *C. elegans* also leads to attached but static chromosome ends. Both mutants show massive defects in synaptonemal complex assembly, suggesting that chromosome movement is additionally involved in synapsis polymerization in *C. elegans* (Sato et al. 2009; Wynne et al. 2012; Zhang et al. 2015). Studies measuring synaptonemal complex polymerization support the notion that chromosome movement promotes extension of the synaptonemal complex, which initiates near to PCs. The pulling force for straightening chromosomes might contribute to the initiation of synaptonemal complex polymerization but also to eliminating unwanted, heterologous chromosome interactions (Rog and Dernburg 2015). The phenotypes of *zyg-12* and dynein-depleted mutants, in which very limited or no synapsis formation is observed, contrast with those of *sun-1(jf18)* or *spd-3(me85)* mutants, in which synapsis formation is delayed but leads to heterologous synapsis or fold-back self-synapsis in the absence of chromosome end movement. Similarly, if the coupling of the chromosome ends to the force generation apparatus is disrupted by deleting the genetic locus encoding all PC proteins, this also leads to delayed, aberrant synapsis (Harper et al. 2011). Finally, studies into the feedback loop regulating SUN-1 phosphorylation also support a role for movement in synaptonemal complex formation (Woglar et al. 2013). In a non-phosphorylatable SUN-1 mutant, SUN-1 aggregates are smaller and the period of SUN-1 aggregate mobility is reduced, particularly under challenged conditions. Synapsis formation is also significantly delayed, suggesting that reduced synaptonemal complex loading might correlate with inefficient force transmission caused by the reduced size of SUN-1 aggregates at chromosome ends (Labella et al. 2011; Woglar et al. 2013).

Aberrant synaptonemal complex formation is also observed in mouse mutants, suggesting a role for telomere attachment and movement in efficient synaptonemal complex assembly here, as well. The severe phenotypes of MAJIN-, TERB1-, and TERB2-deficient mouse strains, where no or limited assembly of synaptonemal central element proteins on chromosomes were observed, demonstrate that telomere attachment is critical to establish synapsis between homologous chromosomes in this species (Shibuya et al. 2015; Shibuya et al. 2014a). SUN1-deficient mice have an intermediate phenotype, perhaps because a significant proportion of their telomeres are attached to the nuclear envelope, which indicates a functional redundancy between SUN1 and SUN2 in this particular process (Ding et al. 2007; Link et al. 2014). Thus, these mice show a less severe phenotype, in which some homologs are fully paired and synapsed, while others are engaged in heterologous associations or unpaired. In mice deficient for KASH5, telomeres attached to the nuclear periphery are not connected to cytoskeletal forces; thus, this mutant might represent a specific deficiency in chromosome movement, without the confounding effects of telomere attachment failure (Horn et al. 2013; Morimoto et al. 2012). Only very limited homologous or heterologous synapsis occurs in KASH5-deficient mice, but whether this is caused by the absence of telomere movement alone is unclear since incomplete telomere attachment was unexpectedly observed. In KASH5^−/−^ mice, 55% telomeres were attached to the nuclear envelope (Horn et al. 2013), which is approximately 15% less than what was observed in SUN1^−/−^ (Link et al. 2014). The less severe synapsis defects in the SUN1^−/−^ mice might suggest that here, any residual movement of attached telomeres might facilitate synapsis formation. Overall, proper telomere attachment appears to function not only in coupling to the movement machinery but also in transmitting a pulling force onto the chromosomes, which could facilitate synaptonemal complex assembly, in both organisms discussed here.

Many movement-defective mutants in both the mouse and *C. elegans* lack not only pairing and synapsis but also show heterologous chromosome associations. These data and studies in other organisms, ranging from plants to yeast, argue that chromosome movement might have a role in preventing or resolving heterologous associations (Koszul and Kleckner 2009; Martinez-Garcia et al. 2018). Mouse mutants with absent or strongly impaired telomere attachment have limited synaptonemal complex formation; thus, heterologous or homologous associations are not stabilized by synapsis (Shibuya et al. 2015, Shibuya et al. 2014a). However, many mutants with distinct types of telomeric defects have extensive synaptonemal complex formation and thus stable chromosome associations. These include mutants in which either a large proportion of telomeres are attached (Ding et al. 2007; Link et al. 2014) or the stability of telomere attachment is impaired (Viera et al. 2009) and those with fully attached telomeres but impaired telomere movement (Link et al. 2013). In many of these mutants, heterologous synapsis is frequently observed. In *C. elegans* mutants in which chromosome ends are attached but movement is impaired often display fold-back-self-synapsis or heterologous synapsis (Labella et al. 2011; Penkner et al. 2007). Chromosomal interlocks are also observed in the worm when the velocity of chromosome end movement is reduced, in particular in the absence of an intact SUN-1 phosphorylation-dependent surveillance system, which would normally prolong the time window of movement (Link et al. 2018). Thus, the phenotypes of movement-defective mutants in both organisms, directly or indirectly, indicate a role for RPMs in resolving undesirable, heterologous interactions and interlocks.

A recent study in *C. elegans* suggests that chromosome movement is also implicated in choosing DSB repair pathways during meiosis (Lawrence et al. 2016). A LINC complex composed of the UNC-84 SUN-domain protein and its KASH partner ZYG-12 promotes DNA repair by homologous recombination. Microtubule poisoning experiments suggested that chromosome motions prevent the use of non-homologous end-joining during meiosis. However, it remains possible that UNC-84 functions in the pathway-choice by directly binding to components of the non-homologous end-joining pathway rather than by regulating chromosome movement (Lawrence et al. 2016).

Several lines of evidence show that telomere attachment and movement is critical for the faithful completion of meiosis in both the mouse and *C. elegans*. Although phenotypes differ in their molecular detail and severity, a general picture is emerging in which the forces exerted through chromosome end attachment are important for (1) efficient and faithful polymerization of the synaptonemal complex exclusively between homologous partners and (2) resolving ectopic and aberrant chromosome interactions. Through this mechanism, RPMs help ensure the efficient side-by-side alignment of homologous chromosomes.

## Concluding remarks and open questions

We have presented detailed information on the timing, general characteristics, and regulation of RPMs in meiotic prophase in two animal models. In both models, RPMs depend on forces generated via microtubules. Synaptonemal complex formation and nuclear envelope restructuring are able to influence and regulate movement characteristics. However, many questions remain.

How is force generated to move chromosomes during leptonema/zygonema? Do chromosomes travel exclusively along existing microtubules and/or is de novo microtubule nucleation required? Immunofluorescence images of the *C. elegans* syncytial gonad reveal a meshwork of microtubules surrounding the nuclei. Their origin is difficult to discern; however, regrowth experiments suggest the plasma membrane as the site of microtubule nucleation (Zhou et al. 2009). Consistent with this, the microtubule nucleator gamma-tubulin localizes to the plasma membrane. However, gamma-tubulin was also detected at the nuclear envelope and at centrosomes, which are still present in this early region of the gonad (Mikeladze-Dvali et al. 2012; Zhou et al. 2009). Do microtubules from pre-meiotic centrosome remnants contribute to chromosome movement? Theoretically, the force for chromosome movement could be generated by either localized microtubule nucleation or motor-dependent movement on existing microtubules. Identifying the site of microtubule nucleation will help us to distinguish between these possibilities. Furthermore, dynein motor proteins play a role in both model organisms. Are kinesin motors also involved? If so, are they needed to change the directionality of the displacement tracks? Investigating the cytoplasmic motor proteins involved in chromosome movement in more detail will provide further insight into the regulation of chromosome movement.

Our understanding of the importance of the highly conserved phenomenon of SUN protein accumulation at the chromosome end attached to the nuclear envelope is still fragmentary. In *C. elegans*, the formation of smaller SUN-1 aggregates correlates with decelerated synaptonemal complex formation. Are SUN-1 aggregates needed to locally concentrate the force involved in straightening the chromosomes and thus to facilitate synaptonemal complex assembly? Or is the locally concentrated force required to oppose other forces that would pull chromosomes ends apart, thereby facilitating assessment of homology to assemble the synaptonemal complex between homologs? How is SUN-1 aggregate formation controlled? What is the stoichiometry of the SUN and KASH proteins within those aggregates? Does kinase activity alone regulate SUN-1 aggregation and, if so, which kinases are involved? This leads to the question of whether we know all movement-relevant CDK, CHK-2, and polo kinase substrates—most likely not.

The mechanism and components involved in meiosis-specific telomere attachment are well described in the mouse. However, the protein factors involved in *C. elegans* chromosome end/PC attachment are less well understood and whether telomeres themselves play a role in the movement and chromosome end attachment process needs to be clarified. Although LINC complex components and several regulatory kinases have been identified in worms, the adaptor proteins linking chromatin–PC–SUN-1 are unknown. An outstanding challenge is to isolate mutants with obvious defects in chromosome end attachment.

Furthermore, the mechanism(s) responsible for moving chromosome ends toward the nuclear periphery at meiotic onset in both models remains largely unclear. It also remains to be investigated which molecular events reorganize chromatin at meiotic onset and in meiotic prophase, when chromosomes are primarily attached to the nuclear envelope through their ends.

In summary, although tremendous novel insights have been achieved in recent years, a lot of important questions still need to be answered.

## References

[CR1] Adelfalk C, Janschek J, Revenkova E, Blei C, Liebe B, Gob E, Alsheimer M, Benavente R, de Boer E, Novak I (2009). Cohesin SMC1beta protects telomeres in meiocytes. J Cell Biol.

[CR2] Alleva B, Smolikove S (2017). Moving and stopping: regulation of chromosome movement to promote meiotic chromosome pairing and synapsis. Nucleus.

[CR3] Alleva B, Balukoff N, Peiper A, Smolikove S (2017). Regulating chromosomal movement by the cochaperone FKB-6 ensures timely pairing and synapsis. J Cell Biol.

[CR4] Alsheimer M, Benavente R (1996). Change of karyoskeleton during mammalian spermatogenesis: expression pattern of nuclear lamin C2 and its regulation. Exp Cell Res.

[CR5] Baudat F, Manova K, Yuen JP, Jasin M, Keeney S (2000). Chromosome synapsis defects and sexually dimorphic meiotic progression in mice lacking Spo11. Mol Cell.

[CR6] Baudrimont A, Penkner A, Woglar A, Machacek T, Wegrostek C, Gloggnitzer J, Fridkin A, Klein F, Gruenbaum Y, Pasierbek P, Jantsch V (2010). Leptotene/zygotene chromosome movement via the SUN/KASH protein bridge in Caenorhabditis elegans. PLoS Genet.

[CR7] Biswas U, Stevense M, Jessberger R (2018). SMC1alpha substitutes for many meiotic functions of SMC1beta but cannot protect telomeres from damage. Curr Biol.

[CR8] Boateng KA, Bellani MA, Gregoretti IV, Pratto F, Camerini-Otero RD (2013). Homologous pairing preceding SPO11-mediated double-strand breaks in mice. Dev Cell.

[CR9] Burke B (2018). LINC complexes as regulators of meiosis. Curr Opin Cell Biol.

[CR10] Cahoon CK, Hawley RS (2013). Flies get a head start on meiosis. PLoS Genet.

[CR11] Chacon MR, Delivani P, Tolic IM (2016). Meiotic nuclear oscillations are necessary to avoid excessive chromosome associations. Cell Rep.

[CR12] Chikashige Y, Ding DQ, Funabiki H, Haraguchi T, Mashiko S, Yanagida M, Hiraoka Y (1994). Telomere-led premeiotic chromosome movement in fission yeast. Science.

[CR13] Chikashige Y, Tsutsumi C, Yamane M, Okamasa K, Haraguchi T, Hiraoka Y (2006). Meiotic proteins bqt1 and bqt2 tether telomeres to form the bouquet arrangement of chromosomes. Cell.

[CR14] Cobb J, Handel MA (1998). Dynamics of meiotic prophase I during spermatogenesis: from pairing to division. Semin Cell Dev Biol.

[CR15] Crisp M, Liu Q, Roux K, Rattner JB, Shanahan C, Burke B, Stahl PD, Hodzic D (2006). Coupling of the nucleus and cytoplasm: role of the LINC complex. J Cell Biol.

[CR16] Daniel K, Trankner D, Wojtasz L, Shibuya H, Watanabe Y, Alsheimer M, Toth A (2014). Mouse CCDC79 (TERB1) is a meiosis-specific telomere associated protein. BMC Cell Biol.

[CR17] Davis L, Smith GR (2006). The meiotic bouquet promotes homolog interactions and restricts ectopic recombination in Schizosaccharomyces pombe. Genetics.

[CR18] de Lange T (2005). Shelterin: the protein complex that shapes and safeguards human telomeres. Genes Dev.

[CR19] Dernburg AF, McDonald K, Moulder G, Barstead R, Dresser M, Villeneuve AM (1998). Meiotic recombination in C. elegans initiates by a conserved mechanism and is dispensable for homologous chromosome synapsis. Cell.

[CR20] Ding X, Xu R, Yu J, Xu T, Zhuang Y, Han M (2007). SUN1 is required for telomere attachment to nuclear envelope and gametogenesis in mice. Dev Cell.

[CR21] Dresser ME (2009). Time-lapse fluorescence microscopy of Saccharomyces cerevisiae in meiosis. Methods Mol Biol.

[CR22] Dresser ME, Giroux CN (1988). Meiotic chromosome behavior in spread preparations of yeast. J Cell Biol.

[CR23] Dunce JM, Milburn AE, Gurusaran M, da Cruz I, Sen LT, Benavente R, Davies OR (2018). Structural basis of meiotic telomere attachment to the nuclear envelope by MAJIN-TERB2-TERB1. Nat Commun.

[CR24] Ellingson DJ, Yao KT (1970). Growth and observations of Chinese hamster seminiferous epithelium in vitro. J Cell Sci.

[CR25] Enguita-Marruedo A, Van Cappellen WA, Hoogerbrugge JW, Carofiglio F, Wassenaar E, Slotman JA, Houtsmuller A, Baarends WM (2018). Live cell analyses of synaptonemal complex dynamics and chromosome movements in cultured mouse testis tubules and embryonic ovaries. Chromosoma.

[CR26] Goldstein P, Slaton DE (1982). The synaptonemal complexes of caenorhabditis elegans: comparison of wild-type and mutant strains and pachytene karyotype analysis of wild-type. Chromosoma.

[CR27] Harper NC, Rillo R, Jover-Gil S, Assaf ZJ, Bhalla N, Dernburg AF (2011). Pairing centers recruit a polo-like kinase to orchestrate meiotic chromosome dynamics in C. elegans. Dev Cell.

[CR28] Herman RK, Kari CK (1989). Recombination between small X chromosome duplications and the X chromosome in Caenorhabditis elegans. Genetics.

[CR29] Herran Y, Gutierrez-Caballero C, Sanchez-Martin M, Hernandez T, Viera A, Barbero JL, de Alava E, de Rooij DG, Suja JA, Llano E (2011). The cohesin subunit RAD21L functions in meiotic synapsis and exhibits sexual dimorphism in fertility. EMBO J.

[CR30] Hiraoka Y, Dernburg AF (2009). The SUN rises on meiotic chromosome dynamics. Dev Cell.

[CR31] Horn HF, Kim DI, Wright GD, Wong ES, Stewart CL, Burke B, Roux KJ (2013). A mammalian KASH domain protein coupling meiotic chromosomes to the cytoskeleton. J Cell Biol.

[CR32] Hughes SE, Miller DE, Miller AL, Hawley RS (2018). Female meiosis: synapsis, recombination, and segregation in Drosophila melanogaster. Genetics.

[CR33] Hunter N (2015) Meiotic recombination: the essence of heredity. Cold Spring Harb Perspect Biol 7 10.1101/cshperspect.a016618 PMC4665078 26511629

[CR34] Ishiguro K, Kim J, Shibuya H, Hernandez-Hernandez A, Suzuki A, Fukagawa T, Shioi G, Kiyonari H, Li XC, Schimenti J, Hoog C, Watanabe Y (2014). Meiosis-specific cohesin mediates homolog recognition in mouse spermatocytes. Genes Dev.

[CR35] Jantsch V, Pasierbek P, Mueller MM, Schweizer D, Jantsch M, Loidl J (2004). Targeted gene knockout reveals a role in meiotic recombination for ZHP-3, a Zip3-related protein in Caenorhabditis elegans. Mol Cell Biol.

[CR36] Keeney S, Giroux CN, Kleckner N (1997). Meiosis-specific DNA double-strand breaks are catalyzed by Spo11, a member of a widely conserved protein family. Cell.

[CR37] Kim Y, Kostow N, Dernburg AF (2015). The chromosome axis mediates feedback control of CHK-2 to ensure crossover formation in C. Elegans. Dev Cell.

[CR38] Klutstein M, Cooper JP (2014). The chromosomal courtship dance-homolog pairing in early meiosis. Curr Opin Cell Biol.

[CR39] Koszul R, Kleckner N (2009). Dynamic chromosome movements during meiosis: a way to eliminate unwanted connections?. Trends Cell Biol.

[CR40] Labella S, Woglar A, Jantsch V, Zetka M (2011). Polo kinases establish links between meiotic chromosomes and cytoskeletal forces essential for homolog pairing. Dev Cell.

[CR41] Labrador L, Barroso C, Lightfoot J, Muller-Reichert T, Flibotte S, Taylor J, Moerman DG, Villeneuve AM, Martinez-Perez E (2013). Chromosome movements promoted by the mitochondrial protein SPD-3 are required for homology search during Caenorhabditis elegans meiosis. PLoS Genet.

[CR42] Lawrence KS, Tapley EC, Cruz VE, Li Q, Aung K, Hart KC, Schwartz TU, Starr DA, Engebrecht J (2016). LINC complexes promote homologous recombination in part through inhibition of nonhomologous end joining. J Cell Biol.

[CR43] Lee CY, Horn HF, Stewart CL, Burke B, Bolcun-Filas E, Schimenti JC, Dresser ME, Pezza RJ (2015). Mechanism and regulation of rapid telomere prophase movements in mouse meiotic chromosomes. Cell Rep.

[CR44] Liebe B, Alsheimer M, Hoog C, Benavente R, Scherthan H (2004). Telomere attachment, meiotic chromosome condensation, pairing, and bouquet stage duration are modified in spermatocytes lacking axial elements. Mol Biol Cell.

[CR45] Liebe B, Petukhova G, Barchi M, Bellani M, Braselmann H, Nakano T, Pandita TK, Jasin M, Fornace A, Meistrich ML, Baarends WM, Schimenti J, de Lange T, Keeney S, Camerini-Otero RD, Scherthan H (2006). Mutations that affect meiosis in male mice influence the dynamics of the mid-preleptotene and bouquet stages. Exp Cell Res.

[CR46] Link J, Jahn D, Schmitt J, Gob E, Baar J, Ortega S, Benavente R, Alsheimer M (2013). The meiotic nuclear lamina regulates chromosome dynamics and promotes efficient homologous recombination in the mouse. PLoS Genet.

[CR47] Link J, Leubner M, Schmitt J, Gob E, Benavente R, Jeang KT, Xu R, Alsheimer M (2014). Analysis of meiosis in SUN1 deficient mice reveals a distinct role of SUN2 in mammalian meiotic LINC complex formation and function. PLoS Genet.

[CR48] Link J, Paouneskou D, Velkova M, Daryabeigi A, Laos T, Labella S, Barroso C, Pacheco Pinol S, Montoya A, Kramer H (2018). Transient and partial nuclear lamina disruption promotes chromosome movement in early meiotic prophase. Dev Cell.

[CR49] Liu L, Franco S, Spyropoulos B, Moens PB, Blasco MA, Keefe DL (2004). Irregular telomeres impair meiotic synapsis and recombination in mice. Proc Natl Acad Sci U S A.

[CR50] Loidl J (1994). Cytological aspects of meiotic recombination. Experientia.

[CR51] Long J, Huang C, Chen Y, Zhang Y, Shi S, Wu L, Liu Y, Liu C, Wu J, Lei M (2017). Telomeric TERB1-TRF1 interaction is crucial for male meiosis. Nat Struct Mol Biol.

[CR52] Lui DY, Colaiacovo MP (2013). Meiotic development in Caenorhabditis elegans. Adv Exp Med Biol.

[CR53] Machovina TS, Mainpal R, Daryabeigi A, McGovern O, Paouneskou D, Labella S, Zetka M, Jantsch V, Yanowitz JL (2016). A surveillance system ensures crossover formation in C. elegans. Curr Biol.

[CR54] MacQueen AJ, Villeneuve AM (2001). Nuclear reorganization and homologous chromosome pairing during meiotic prophase require C. elegans chk-2. Genes Dev.

[CR55] MacQueen AJ, Colaiacovo MP, McDonald K, Villeneuve AM (2002). Synapsis-dependent and -independent mechanisms stabilize homolog pairing during meiotic prophase in C. elegans. Genes Dev.

[CR56] MacQueen AJ, Phillips CM, Bhalla N, Weiser P, Villeneuve AM, Dernburg AF (2005). Chromosome sites play dual roles to establish homologous synapsis during meiosis in C. elegans. Cell.

[CR57] Martinez-Garcia M, Schubert V, Osman K, Darbyshire A, Sanchez-Moran E, Franklin FCH (2018). TOPII and chromosome movement help remove interlocks between entangled chromosomes during meiosis. J Cell Biol.

[CR58] McKim KS, Howell AM, Rose AM (1988). The effects of translocations on recombination frequency in Caenorhabditis elegans. Genetics.

[CR59] McKim KS, Peters K, Rose AM (1993). Two types of sites required for meiotic chromosome pairing in Caenorhabditis elegans. Genetics.

[CR60] Mikeladze-Dvali T, von Tobel L, Strnad P, Knott G, Leonhardt H, Schermelleh L, Gonczy P (2012). Analysis of centriole elimination during C. elegans oogenesis. Development.

[CR61] Mikolcevic P, Isoda M, Shibuya H, del Barco Barrantes I, Igea A, Suja JA, Shackleton S, Watanabe Y, Nebreda AR (2016). Essential role of the Cdk2 activator RingoA in meiotic telomere tethering to the nuclear envelope. Nat Commun.

[CR62] Morelli MA, Werling U, Edelmann W, Roberson MS, Cohen PE (2008). Analysis of meiotic prophase I in live mouse spermatocytes. Chromosom Res.

[CR63] Morimoto A, Shibuya H, Zhu X, Kim J, Ishiguro K, Han M, Watanabe Y (2012). A conserved KASH domain protein associates with telomeres, SUN1, and dynactin during mammalian meiosis. J Cell Biol.

[CR64] Parvinen M, Soderstrom KO (1976). Chromosome rotation and formation of synapsis. Nature.

[CR65] Pendlebury DF, Fujiwara Y, Tesmer VM, Smith EM, Shibuya H, Watanabe Y, Nandakumar J (2017). Dissecting the telomere-inner nuclear membrane interface formed in meiosis. Nat Struct Mol Biol.

[CR66] Penkner A, Tang L, Novatchkova M, Ladurner M, Fridkin A, Gruenbaum Y, Schweizer D, Loidl J, Jantsch V (2007). The nuclear envelope protein Matefin/SUN-1 is required for homologous pairing in C. elegans meiosis. Dev Cell.

[CR67] Penkner AM, Fridkin A, Gloggnitzer J, Baudrimont A, Machacek T, Woglar A, Csaszar E, Pasierbek P, Ammerer G, Gruenbaum Y, Jantsch V (2009). Meiotic chromosome homology search involves modifications of the nuclear envelope protein Matefin/SUN-1. Cell.

[CR68] Peoples-Holst TL, Burgess SM (2005). Multiple branches of the meiotic recombination pathway contribute independently to homolog pairing and stable juxtaposition during meiosis in budding yeast. Genes Dev.

[CR69] Phillips CM, Dernburg AF (2006). A family of zinc-finger proteins is required for chromosome-specific pairing and synapsis during meiosis in C. elegans. Dev Cell.

[CR70] Phillips CM, Wong C, Bhalla N, Carlton PM, Weiser P, Meneely PM, Dernburg AF (2005). HIM-8 binds to the X chromosome pairing center and mediates chromosome-specific meiotic synapsis. Cell.

[CR71] Phillips CM, Meng X, Zhang L, Chretien JH, Urnov FD, Dernburg AF (2009). Identification of chromosome sequence motifs that mediate meiotic pairing and synapsis in C. Elegans. Nat Cell Biol.

[CR72] Rog O, Dernburg AF (2015) Direct visualization reveals kinetics of meiotic chromosome synapsis. Cell Rep 10.1016/j.celrep.2015.02.032 PMC4565782 25772351

[CR73] Romanienko PJ, Camerini-Otero RD (2000). The mouse Spo11 gene is required for meiotic chromosome synapsis. Mol Cell.

[CR74] Rose AM, Baillie DL, Curran J (1984). Meiotic pairing behavior of two free duplications of linkage group I in Caenorhabditis elegans. Mol Gen Genet.

[CR75] Rosenbluth RE, Baillie DL (1981). The genetic analysis of a reciprocal translocation, eT1(III; V), in Caenorhabditis elegans. Genetics.

[CR76] Salonen K, Paranko J, Parvinen M (1982). A colcemid-sensitive mechanism involved in regulation of chromosome movements during meiotic pairing. Chromosoma.

[CR77] Sanford C, Perry MD (2001). Asymmetrically distributed oligonucleotide repeats in the Caenorhabditis elegans genome sequence that map to regions important for meiotic chromosome segregation. Nucleic Acids Res.

[CR78] Sato A, Isaac B, Phillips CM, Rillo R, Carlton PM, Wynne DJ, Kasad RA, Dernburg AF (2009). Cytoskeletal forces span the nuclear envelope to coordinate meiotic chromosome pairing and synapsis. Cell.

[CR79] Scherthan H, Adelfalk C (2011). Live cell imaging of meiotic chromosome dynamics in yeast. Methods Mol Biol.

[CR80] Scherthan H, Weich S, Schwegler H, Heyting C, Harle M, Cremer T (1996). Centromere and telomere movements during early meiotic prophase of mouse and man are associated with the onset of chromosome pairing. J Cell Biol.

[CR81] Schmitt J, Benavente R, Hodzic D, Hoog C, Stewart CL, Alsheimer M (2007). Transmembrane protein Sun2 is involved in tethering mammalian meiotic telomeres to the nuclear envelope. Proc Natl Acad Sci U S A.

[CR82] Shibuya H, Watanabe Y (2014). The meiosis-specific modification of mammalian telomeres. Cell Cycle.

[CR83] Shibuya H, Ishiguro K, Watanabe Y (2014). The TRF1-binding protein TERB1 promotes chromosome movement and telomere rigidity in meiosis. Nat Cell Biol.

[CR84] Shibuya H, Morimoto A, Watanabe Y (2014). The dissection of meiotic chromosome movement in mice using an in vivo electroporation technique. PLoS Genet.

[CR85] Shibuya H, Hernandez-Hernandez A, Morimoto A, Negishi L, Hoog C, Watanabe Y (2015). MAJIN links telomeric DNA to the nuclear membrane by exchanging telomere cap. Cell.

[CR86] Tang X, Jin Y, Cande WZ (2006). Bqt2p is essential for initiating telomere clustering upon pheromone sensing in fission yeast. J Cell Biol.

[CR87] Trelles-Sticken E, Loidl J, Scherthan H (1999). Bouquet formation in budding yeast: initiation of recombination is not required for meiotic telomere clustering. J Cell Sci.

[CR88] Tu Z, Bayazit MB, Liu H, Zhang J, Busayavalasa K, Risal S, Shao J, Satyanarayana A, Coppola V, Tessarollo L, Singh M, Zheng C, Han C, Chen Z, Kaldis P, Gustafsson JÅ, Liu K (2017). Speedy A-Cdk2 binding mediates initial telomere-nuclear envelope attachment during meiotic prophase I independent of Cdk2 activation. Proc Natl Acad Sci U S A.

[CR89] Viera A, Rufas JS, Martinez I, Barbero JL, Ortega S, Suja JA (2009). CDK2 is required for proper homologous pairing, recombination and sex-body formation during male mouse meiosis. J Cell Sci.

[CR90] Viera A, Alsheimer M, Gomez R, Berenguer I, Ortega S, Symonds CE, Santamaria D, Benavente R, Suja JA (2015). CDK2 regulates nuclear envelope protein dynamics and telomere attachment in mouse meiotic prophase. J Cell Sci.

[CR91] Villeneuve AM (1994). A cis-acting locus that promotes crossing over between X chromosomes in Caenorhabditis elegans. Genetics.

[CR92] Wang L, Tu Z, Liu C, Liu H, Kaldis P, Chen Z, Li W (2018). Dual roles of TRF1 in tethering telomeres to the nuclear envelope and protecting them from fusion during meiosis. Cell Death Differ.

[CR93] Wang Y, Chen Y, Chen J, Wang L, Nie L, Long J, Chang H, Wu J, Huang C, Lei M (2019). The meiotic TERB1-TERB2-MAJIN complex tethers telomeres to the nuclear envelope. Nat Commun.

[CR94] Woglar A, Jantsch V (2014). Chromosome movement in meiosis I prophase of Caenorhabditis elegans. Chromosoma.

[CR95] Woglar A, Villeneuve AM (2018). Dynamic architecture of DNA repair complexes and the synaptonemal complex at sites of meiotic recombination. Cell.

[CR96] Woglar A, Daryabeigi A, Adamo A, Habacher C, Machacek T, La Volpe A, Jantsch V (2013). Matefin/SUN-1 phosphorylation is part of a surveillance mechanism to coordinate chromosome synapsis and recombination with meiotic progression and chromosome movement. PLoS Genet.

[CR97] Wynne DJ, Rog O, Carlton PM, Dernburg AF (2012). Dynein-dependent processive chromosome motions promote homologous pairing in C. elegans meiosis. J Cell Biol.

[CR98] Yao KT, Ellingson DJ (1969). Observations on nuclear rotation and oscillation in Chinese hamster germinal cells in vitro. Exp Cell Res.

[CR99] Zeng X, Li K, Yuan R, Gao H, Luo J, Liu F, Wu Y, Wu G, Yan X (2017). Nuclear envelope-associated chromosome dynamics during meiotic prophase I. Front Cell Dev Biol.

[CR100] Zhang L, Ward JD, Cheng Z, Dernburg AF (2015). The auxin-inducible degradation (AID) system enables versatile conditional protein depletion in C. elegans. Development.

[CR101] Zhang J, Tu Z, Watanabe Y, Shibuya H (2017). Distinct TERB1 domains regulate different protein interactions in meiotic telomere movement. Cell Rep.

[CR102] Zhou K, Rolls MM, Hall DH, Malone CJ, Hanna-Rose W (2009). A ZYG-12-dynein interaction at the nuclear envelope defines cytoskeletal architecture in the C. elegans gonad. J Cell Biol.

[CR103] Zickler Denise, Kleckner Nancy (2015). Recombination, Pairing, and Synapsis of Homologs during Meiosis. Cold Spring Harbor Perspectives in Biology.

[CR104] Zickler D, Kleckner N (2016). A few of our favorite things: pairing, the bouquet, crossover interference and evolution of meiosis. Semin Cell Dev Biol.

